# Biomarkers of Response to Etoposide-Platinum Chemotherapy in Patients with Grade 3 Neuroendocrine Neoplasms

**DOI:** 10.3390/cancers13040643

**Published:** 2021-02-05

**Authors:** Caroline Lacombe, Ophélie De Rycke, Anne Couvelard, Anthony Turpin, Aurélie Cazes, Olivia Hentic, Valérie Gounant, Gérard Zalcman, Philippe Ruszniewski, Jérôme Cros, Louis de Mestier

**Affiliations:** 1 Université de Paris, Department of Gastroenterology-Pancreatology, ENETS Centre of Excellence, Beaujon University Hospital (APHP), 92110 Clichy, France; lacombe_caroline@hotmail.fr (C.L.); opheliederycke@wanadoo.fr (O.D.R.); olivia.hentic@aphp.fr (O.H.); philippe.ruszniewski@aphp.fr (P.R.); 2 Université de Paris, Centre of Research on Inflammation, INSERM U1149, 75018 Paris, France; anne.couvelard@aphp.fr (A.C.); jerome.cros@aphp.fr (J.C.); 3 Université de Paris, Department of Pathology, ENETS Centre of Excellence, Beaujon/Bichat University Hospital (APHP), 75018 Paris, France; aurelie.cazes@aphp.fr; 4 Department of Medical Oncology, Claude Huriez University Hospital, 59000 Lille, France; anthony.turpin@chru-lille.fr; 5 Université de Paris, Department of Thoracic Oncology, CIC INSERM 1425, Bichat University Hospital, 75018 Paris, France; valerie.gounant@aphp.fr (V.G.); gerard.zalcman@aphp.fr (G.Z.)

**Keywords:** neuroendocrine neoplasms, chemotherapy, biomarkers, tumor response

## Abstract

**Simple Summary:**

Grade 3 neuroendocrine neoplasms (G3 NEN) are a heterogenous subtype of NEN, including well-differentiated neuroendocrine tumors (G3 NET) and poorly differentiated large- or small-cell type carcinomas (NEC). Until recently, all G3 NEN were considered to be poorly differentiated NEN and were treated with etoposide-platinum (EP) chemotherapy, which is usually used in pulmonary NEC. However, G3 NET and NEC have a different prognosis and response to EP chemotherapy, which is usually poor in G3 NET. The aim of this study was to evaluate the predictive biomarkers of response (Rb, p53 and p16) to EP chemotherapy in patients with G3 NEN reviewed by two expert pathologists. Identifying the predictive biomarkers of the response to EP treatment could help oncologists identify the subgroup of patients which could benefit from EP therapy and offer more personalized treatment.

**Abstract:**

Etoposide-platinum (EP) chemotherapy has long been the reference treatment for grade 3 neuroendocrine neoplasms (G3 NEN). However, G3 NEN are heterogeneous, including well-differentiated tumors (NET) and poorly differentiated large (LCNEC) or small (SCNEC) cell carcinomas, whose response to EP chemotherapy varies considerably. Our aim was to evaluate predictive biomarkers for the response to EP chemotherapy in G3 NEN. We retrospectively studied 89 patients with lung (42%) and digestive (58%) G3 NEN treated by EP chemotherapy between 2006 and 2020. All cases were centrally reviewed for cytomorphology/Ki-67 and immunohistochemistry of retinoblastoma protein (Rb)/p53/p16, analyzed using a semi-quantitative score. The absence of Rb staining (Rb^inap^) or the absence of very intense p53 staining (p53^inap^) were considered inappropriate. Rb staining was also studied as a quantitative marker, the best threshold being determined by ROC curve. Intense p16 staining (p16^high^) also suggested cell cycle dysregulation. Our primary endpoint was the objective response rate (ORR). We included 10 G3 NET, 31 LCNEC and 48 SCNEC, which showed ORR of 20%, 32% and 75%, respectively (NET vs. NEC, *p* = 0.040; LCNEC vs. SCNEC, *p* < 0.001). The ORR was significantly higher in NEN presenting with Rb^inap^ (63% vs. 42%, *p* = 0.025) and p16^high^ (66% vs. 35%, *p* = 0.006). Rb < 150 optimally identified responders (AUC = 0.657, *p* < 0.001). The ORR was 67% in Rb < 150 (vs. 25%, *p* = 0.005). On multivariate analysis, only Rb < 150 was independently associated with ORR (OR 4.16, 95% CI 1.11–15.53, *p* = 0.034). We confirm the heterogeneity of the response to EP treatment in G3 NEN. Rb < 150 was the best predictive biomarker for the response to EP, and p53 immunostaining had no additional value.

## 1. Introduction

Neuroendocrine neoplasms (NEN) are a rare type of tumor with a heterogeneous prognosis, that mainly arise from the gastrointestinal tract or the lungs. The management and prognosis of patients with NEN depend, among other factors, on the WHO classification [1,2], which is based on the morphological differentiation (well-differentiated neuroendocrine tumors (NET) or poorly differentiated neuroendocrine carcinomas (NEC)). NEC are characterized as large-cell (LCNEC) or small-cell (SCNEC) NEC depending on their cell morphology. Moreover, digestive NEN are subclassified according to their grade (G) which depends on the Ki-67 proliferative index and/or the mitotic count: G1 (Ki-67 < 3%), G2 (Ki-67 between 3% and 20%) or G3 (Ki67 > 20%) [1]. Lung NEN are classified as well-differentiated typical (mitotic count <2/mm^2^ without necrosis) or atypical (2–10/mm^2^ with focal necrosis) carcinoids, or NEC (mitotic count >10/mm^2^ with extensive necrosis) [2]. Until recently, all high-grade (G3) NEN were considered to be poorly differentiated NEC and treated with etoposide-platinum (EP) chemotherapy like lung SCNEC [3]. 

However, high-grade or G3 NEN are a heterogeneous group. Indeed, while all of these tumors were initially considered to be poorly differentiated NEC, evidence of a subgroup of well-differentiated high-grade or G3 NET has increased in the last decade. High-grade lung NEN with carcinoid morphology have recently been described, and were recognized as an entity that does not fit into any of the existing groups of the WHO classification. These pulmonary NEN are characterized by a combination of a well-differentiated morphology and a high proliferation rate with a mitotic count >10/2 mm^2^ [4,5]. In addition, different molecular subtypes of lung LCNEC have been described, with an SCLC-like subtype defined by the co-existence of retinoblastoma protein (Rb) loss and *TP53* mutations without *KRAS* or *STK11* mutations, and an adenocarcinoma-like subtype defined by the presence of *KRAS* or *STK11* mutations [6,7,8,9,10]. Similarly, pancreatic G3 NET were described as a specific entity in the 2017 WHO classification of endocrine NEN [11], which was further extended to all digestive NEN in 2019 [1]. Like their G1 and G2 counterparts, pancreatic G3 NET carry *DAXX* and *ATRX* mutations (around 40%) with rare alterations of *RB1* and *TP53*. Conversely, NEC frequently present Rb pathway dysregulation (36–86%), and/or mutations in *TP53* (62–95%) [12,13,14,15,16]. 

Besides classifications and biomarkers, patients with G3 NET also have a better prognosis than those with NEC, although they show a decreased response to EP chemotherapy [13,17,18,19,20,21]. Nevertheless, no reliable predictive biomarkers of the efficacy of EP treatment in G3 NEN have been clearly identified. Rb and p16, two major cell cycle regulators, play a role in the pathogenesis of NEC. Dysregulation of the Rb pathway has been shown to occur through Rb loss or amplification of cyclin genes, all resulting in overexpression of p16 in NEC [16,22,23]. Moreover, alterations in the p53-encoding gene *TP53* is one of the most frequent genetic alterations in pulmonary NEC and have also been described in digestive NEC [7,14,24,25,26]. These molecular mechanisms suggest that Rb, p16 and p53 could be potential prognostic factors. Recent studies suggested that Rb loss and/or p16 overexpression could be associated with prolonged OS under EP chemotherapy in pulmonary LCNEC (SCLC-like subtype) [10,27] and an increased response to EP therapy in pancreatic G3 NEN [28,29]. 

The aim of our study was to explore predictive biomarkers for a morphological response to EP treatment in lung and digestive G3 NEN.

## 2. Results

### 2.1. Patient Characteristics

We identified 153 patients with NEN who received EP chemotheraphy from January 2006 to March 2020 at Beaujon Hospital. We excluded 34 patients after a clinical and pathological review and 30 additional patients because immunohistochemistry (IHC) was not feasible; thus, 89 patients were included in the final analysis (Figure S1). Briefly, the median age was 61.6 years old with a male predominance (sex-ratio 2:1) and most patients had a performance status of 0 or 1. Digestive primary NEN was identified in 58% of cases (Table 1). Most patients (*n* = 71, 80%) had metastases with hepatic localizations in the majority.

Overall, 10, 31 and 48 patients had NEN categorized as G3 NET, LCNEC or SCNEC, with median Ki-67 indexes of 30% (26.8–39), 70% (50–90) and 90% (80–95), respectively. Following double pathological central review, the NEN classification was changed for 12/89 cases (13.5%) compared with initial diagnosis (as specified in patient records). Digestive and lung NEN accounted for 100% and 0% of G3 NET, 77% and 23% of LCNEC and 38% and 63% of SCNEC, respectively (Table S1). The median Ki-67 index was 90% and 70% in pulmonary and digestive NEN, respectively.

Patients underwent a median of six cycles (4–7) of EP chemotherapy that combined etoposide with cisplatin or carboplatin in 18% and 82% of cases, respectively. This chemotherapy was first line in 93.3% of patients, although less frequently among G3 NET patients (*n* = 3/10, 30%), including six patients who received it as neoadjuvant treatment. The six remaining patients (5/6 pancreatic NEN; 3 G3 NET, 2 LCNEC and 1 SCNEC, median Ki67 80%) received EP chemotherapy in second line. The toxicity of EP chemotherapy was comparable to previous studies (Table S2).

### 2.2. Biomarker Immunohistochemistry

**Figure 1:**
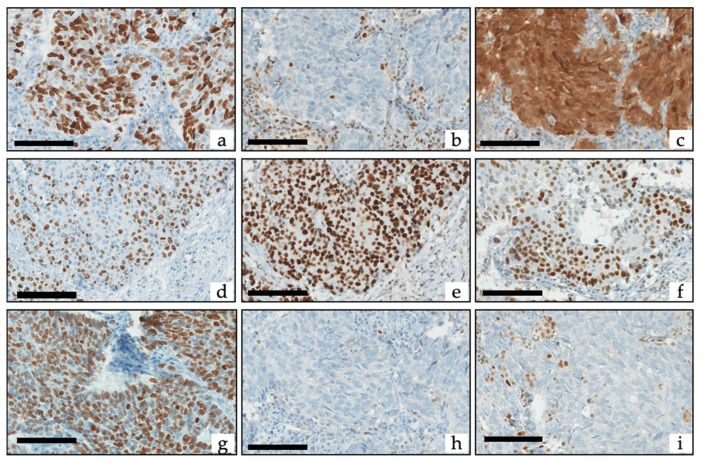
Example of Ki-67, retinoblastoma protein (Rb), p16 and p53 immunohistochemistry: (**a**–**c**) SCNEC with (**a**) Ki-67 at 90%, (**b**) loss of Rb (with positive internal controls) corresponding to the absence of Rb staining (Rb^inap^) pattern, and (**c**) high expression of p16 corresponding to the intense p16 staining (p16^high^) pattern. (**d**–**f**) LCNEC with (**d**) Ki-67 at 60%, (**e**) diffuse overexpression of p53 corresponding to the inappropriate p53 staining (p53^inap^) pattern, and (**f**) conserved expression of Rb corresponding to the appropriate Rb staining (Rb^app^) pattern. (**g**–**i**) SCNEC with (**a**) Ki-67 at 83%, (**h**) complete loss of p53 (with positive internal controls) corresponding to the p53^inap^ pattern, and (**i**) loss of Rb (with positive internal controls) corresponding to the Rb^inap^ pattern. Scale bars correspond to 50 µm.

**Figure 2:**
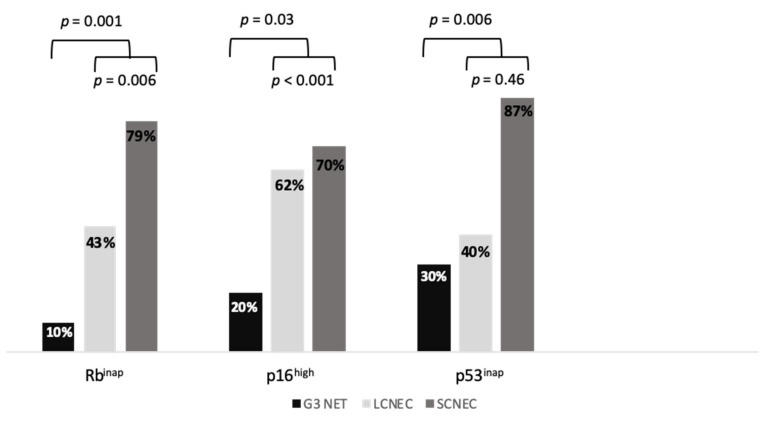
Rb, p53 and p16 status in G3 NET, LCNEC and SCNEC. LCNEC, large-cell neuroendocrine carcinoma; NET, neuroendocrine tumor; SCNEC, small-cell neuroendocrine carcinoma.

Tumor samples included biopsies or surgical specimens in 82 and 7 patients, respectively. Rb, p16 and p53 immunostaining could not be performed in two, two and three patients, respectively. The absence of Rb staining (Rb^inap^) was detected in 1/10 G3 NET (10%), 13/30 LCNEC (43.3%) and 37/47 SCNEC (78.7%) patients (Figure 1). The proportion of Rb^inap^ was higher in SCNEC than in LCNEC (*p* = 0.006) and higher in NEC than in NET (*p* = 0.001) (Figure 2). The proportion of Rb^inap^ was 78.3% and 44% in lung and digestive NEN, respectively (Table S1). Among NEC, SCNEC were more likely to be Rb^inap^ than LCNEC (OR 3.33, 95% CI (1.14–9.71), *p* = 0.027), independent from their origin in the lung (OR 1.93, 95% CI (0.65–5.77), *p* = 0.237). 

Intense p16 staining (p16^high^) was detected in 3/10 G3 NET (30%), 12/30 LCNEC (40%) and 41/47 SCNEC (87.2%) patients. The proportion of p16^high^ was higher in SCNEC than in LCNEC (*p* < 0.001) and higher in NEC than in NET (*p* = 0.03) (Figure 2). The proportion of p16^high^ was 86.1% and 49% in lung and digestive NEN, respectively (Table S1). SCNEC were more likely to be p16^high^ than LCNEC (OR 7.29, 95% CI (2.22–23.93), *p* = 0.001), independent of their origin in the lung (OR 2.71, 95% CI (0.77–9.57)), *p* = 0.123). Most G3 NEN were classified as Rb^inap^/p16^high^ (52.9%) or appropriate Rb staining (Rb^app^)/p16^low^ (28.2%), while a minority were classified as Rb^app^/p16^high^ (12.9%) or Rb^inap^/p16^low^ (5.9%). There was an inverse correlation between Rb and p16 scoring (rho= −0.62, *p* < 0.001). 

Inappropriate p53 staining (p53^inap^) was detected in 2/10 G3 NET (20%), 18/29 LCNEC (62.1%) and 33/47 SCNEC (70.2%) patients. The proportion of p53^inap^ was similar in SCNEC and in LCNEC (*p* = 0.46) and was higher in NEC than in NET (*p* = 0.006) (Figure 2). The proportion of p53^inap^ was 67.6% and 57.1% in lung and digestive NEN, respectively (Table S1).

### 2.3. Association between Biomarkers and Response to EP

**Figure 3:**
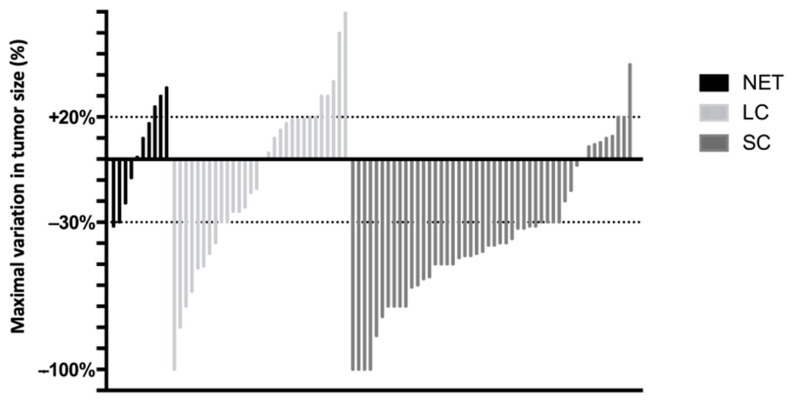
Waterfall plot of maximal variation (%) in tumor size compared to baseline scans, determined by the Response Evaluation Criteria in Solid Tumors (RECIST) method. The dotted lines correspond to the thresholds for progression (+20%) or an objective response (−30%). LCNEC, large-cell neuroendocrine carcinoma; NET, neuroendocrine tumor; SCNEC, small-cell neuroendocrine carcinoma.

The objective response rate (ORR) to EP chemotherapy was 20% (2/10), 32% (10/31) and 75% (36/48) in patients with G3 NET, LCNEC and SCNEC, respectively (Figure 3). The ORR was significantly higher in SCNEC than in LCNEC (*p* < 0.001) and in NEC than in G3 NET (*p* = 0.04). The disease control rate was 60%, 71% and 94%, respectively. This rate was significantly higher in SCNEC than in LCNEC (*p* = 0.009) and higher in NEC than in G3 NET (*p* = 0.08). 

**Figure 4:**
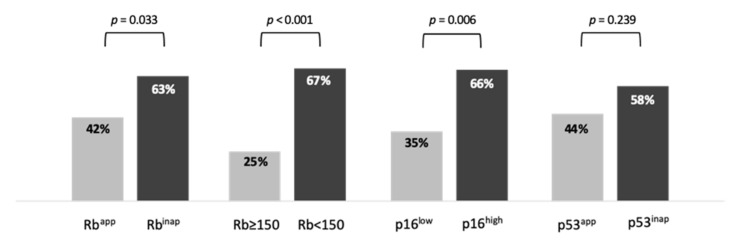
Response rate depending on biomarker expression.

The ORR was significantly higher in Rb^inap^ (33/51, 63%) than in Rb^app^ NEN (15/36, 42%, *p* = 0.033) (Figure 4). Because Rb immunostaining was evaluated using a semi-quantitative score (0 to 300), we also analyzed Rb expression as a quantitative variable (Figure S2). The optimal cut-off value to distinguish between responders and non-responders was Rb = 150 (AUC = 0.656, *p* = 0.012). The ORR was significantly higher in NEN with a Rb score <150 (42/63, 67%) than in those with an Rb score ≥150 NEN (6/24, 25%, *p* < 0.001). The ORR was also higher in p16^high^ (37/56, 66%) than in p16^low^ NEN (11/31, 35%, *p* = 0.006) (Figure 4). Finally, the ORR was 58% in p53^inap^ NEN and 44% in p53^app^ NEN (*p* = 0.239).

In multivariate logistic regression analysis, an Rb score <150 was significantly associated with an objective response (OR 4.16, 95% CI (1.11–15.53), *p* = 0.034), after adjustment for primary tumor location (*p* = 0.57), WHO classification (*p* = 0.001) and Ki-67 (*p* = 0.31) (Table 2). On the other hand, Rb^inap^ (OR 1.7, 95% CI (0.54–5.37), *p* = 0.368) and p16^high^ (OR 1.61, 95% CI (0.47–5.56), *p* = 0.449) were not associated with an objective response after adjustment for the same prognostic variables. SCNEC remained strongly and significantly associated with an objective response in both multivariate models (Table 2).

Among the six patients who received second-line EP, only one patient had an objective response (pancreatic SCNEC with Rb^inap^, p16^low^, p53^inap^), giving a response rate of 16.7%.

### 2.4. Association between Biomarkers and PFS under EP

Progression-free survival (PFS) following EP treatment could be evaluated in all patients. Median PFS was 6.89 months (95% CI (0.18–13.6)), 4.56 months (95% CI (3.12–6)) and 6.36 months (95% CI (5.16–7.56)) in patients with G3 NET, LCNEC and SCNEC, respectively. In the subgroup of patients with NEC, an Rb score <150 (HR 0.37, 95% CI (0.17–0.8), *p* = 0.012) and p16^high^ (HR 0.39, 95% CI (0.19–0.79), *p* = 0.009), were both independently associated with a significantly lower risk of progression on multivariate analysis adjusted for primary tumor location, NEC subtype, Ki-67 and extra-hepatic metastases (Table 3). Conversely, Rb^inap^ only influenced the PFS but this was not statistically significant (HR 0.57, 95% CI (0.32–1.02), *p* =0.058).

### 2.5. Association between Biomarkers and OS

Median OS was 17.21 months (95% CI (13.59–20.83)), 9.97 months (95% CI (6.81–13.3)) and 8.3 months (95% CI (7.02–9.58)) in patients with G3 NET, LCNEC and SCNEC, respectively. In the subgroup of patients with NEC, Rb^inap^ was independently associated with a significantly lower risk of death (HR 0.54, 95% CI (0.31–0.95), *p* = 0.033) on multivariate analyses adjusted for primary tumor location, NEC subtype, Ki-67 and extra-hepatic metastases (Table 4). Conversely, an Rb score <150 (HR 0.50, 95% CI (0.25–1.01), *p* = 0.053) or p16^high^ (HR 0.52, 95% CI (0.26–1.05), *p* = 0.067) only influenced OS but was not statistically significant. 

## 3. Discussion

Here, we report that Rb and p16 are reliable predictive biomarkers of an objective response to EP chemotherapy in G3 NEN. While the ORR was higher in patients with Rb^inap^ NEN, prediction of ORR by Rb <150 was more relevant and independent of other traditional factors. 

Our study confirms the heterogeneity of the molecular features of G3 NEN. Inappropriate Rb, p16 and/or p53 expression were more frequent in NEC in comparison with NET; they were also more common in SCNEC in comparison with LCNEC, in accordance with the literature [23,24,30]. Due to the lack of well-defined cyto-morphological criteria, the above-mentioned biomarkers could help classify G3 NEN [12,31]. It is essential to distinguish between G3 NET and NEC because these patients have significantly different prognosis and response to EP chemotherapy. In our cohort, the ORR was significantly higher in NEC than in G3 NET treated with EP chemotherapy (58% vs. 20%, *p* = 0.04). In previous studies, the ORR to EP treatment varied from 0% to 17% in digestive G3 NET, from 30% to 56% in digestive NEC [18,19,29,32] and 33% to 86% in lung NEC [30,33], showing the importance of differentiating NET from NEC to predict the efficacy of EP. Furthermore, the ORR to EP therapy in patients with NEC is higher in those with SCNEC than in those with LCNEC. The ORR in a series of lung NEC ranged from 57% to 86% in patients with SCNEC and from 33% to 73% in those with LCNEC [10,34,35]. This was also reported in patients with pancreatic NEC in the study by Hijioka et al. [28] in which the ORR was 57% and 36% in those with SCNEC and LCNEC, respectively. Accordingly, in the present study, SCNEC was associated with a significantly higher ORR (75% vs. 32% in LCNEC; *p* < 0.001), which remained statistically significant on multivariate analysis (*p* = 0.001). 

Although SCNEC appears to be strongly predictive of the efficacy of EP, the morphological distinction with LCNEC is still a challenge for pathologists, with an agreement of only 40–70% [32,34]. Thus, identifying biomarkers predictive of EP treatment efficacy in G3 NEC would be of clear interest, and Rb appears to be a relevant candidate. Rb is involved in the S-phase checkpoint and is more frequently altered in NEC that respond to EP. Our study showed that the ORR was significantly higher in Rb^inap^ G3 NEN (63%, vs. 42% in Rb^app^; *p* = 0.033). This is similar to a previous study which reported an ORR of 71% (12/17) and 21% (5/24) in Rb-deficient and Rb-proficient pancreatic G3 NEN, respectively (*p* = 0.003) [28]. However, in our study Rb^inap^ was no longer associated with a higher ORR in multivariate analysis. We therefore reevaluated our method of analyzing the Rb deficiency in G3 NEN. Rb is usually reported as a binary value (loss/retained), although most studies used a quantitative score with various thresholds to define deficiency (H-score < 50/300 [27], H-score < 40/300 [22], <80% of positive cells [36], negative staining [12,29,33]). When we analyzed the association between Rb expression as a quantitative variable and an objective response, we found that a score <150 was the optimal threshold to significantly distinguish between responders and non-responders. The ORR was significantly higher in NEN with an Rb score <150 (67%) than in those with an Rb score ≥150 (25%, *p* < 0.001). Most interestingly and unlike Rb^inap^, an Rb score <150 remained independently associated with a significantly higher probability of an objective response on multivariate analysis (*p* = 0.034), in particular when adjusted for SCNEC histology (Table 2, model 2). Hence, Rb < 150 appeared to be the best predictive biomarker of a response to EP chemotherapy in G3 NEN. This is also shown by its significant association with a prolonged PFS, as Rb < 150 was associated with a 63% reduction in the risk of progression (95% CI, 0.17–0.8, *p* = 0.012) independent from other usual prognostic factors. Finally, Rb < 150 was also associated with prolonged survival in patients with G3 NEN treated with EP, although this was not significant on multivariate analysis (HR 0.50, 95% CI 0.25–1.01; *p* = 0.053). This is similar to the results by Derks et al. [27] showing that OS was higher in mutated *RB1* or Rb <50 in a group of patients with lung LCNEC treated with EP. This suggests that in addition to Rb complete inactivation (loss of expression), other mechanisms of partial Rb silencing may also be at play and impact response to EP.

The ORR was significantly higher in patients with p16^high^ G3 NEN (66%, vs. 35% in p16^low^; *p* = 0.006). Like Rb, p16 is another G1/S transition regulator of the cell cycle which inactivates cyclin-dependent kinases that phosphorylate Rb. It has been reported that Rb was inversely correlated with p16 in 90% and 62% of lung and digestive NEC, respectively, probably because Rb loss results in p16 upregulation [16,22]. However, in our study p16^high^ was not significantly associated with ORR in multivariate analysis, (Table 2, model 3). This is not surprising since p16^high^ has been found to be more strongly correlated to SCNEC than to other histological subgroups of lung and digestive NEC [35,37]. Thus, in our cohort, p16^high^ was the biomarker that most strongly correlated to SCNEC (Figure 2). This suggests that it may be predictive of a higher ORR with EP chemotherapy as a surrogate of SCNEC histology. 

Our results showed that the p53 status did not influence ORR. One hypothesis is that p53 expression does not account for all *TP53* gene alterations. It has been demonstrated that the correlation between *TP53* gene mutation and p53 protein expression in tumor cells was only 70% based on studies analyzing the entire *TP53* gene. This relationship could be influenced by the site of the mutation, the resulting substitution and some natural polymorphisms [38]. Hence, p53 immunohistochemical expression might be used as a screening tool but is not sufficiently sensitive to detect all different types of mutations and that performing sequencing could be a solution [39].

The main limitation of this study is its retrospective design. However, there was a low proportion of missing data. In addition, although this was a single-center series it was performed in a high-volume center with long-term expertise in NEN, and all G3 NEN cases were centrally reviewed by two expert pathologists. The G3 NET subgroup was smaller than the NEC subgroup, but G3 NET are rare. Since G3 NET were identified as a distinct category, they have no longer been treated like NEC in our center. Thus, the period of treatment for G3 NET may precede that for NEC, which could influence overall survival (OS) analyses but not the ORR, which was our primary endpoint. In addition, while G3 NET and NEC have marked survival differences, this, like the somewhat long inclusion period, did not influence the analysis of objective response. Our population included both digestive and pulmonary NEN, but analyses were adjusted for the primary location. Finally, while we investigated immunostaining markers linked to molecular features of NEN, we did not investigate the molecular features themselves, as underlined above for p53. However, immunohistochemistry presents the advantages of being a cheap, reproducible and readily available technique, and potentially useful as shown in this study.

Our results indicate that patients with NEC with Rb > 150 and/or p16^low^ may not be good candidates for EP chemotherapy. In this subgroup, mostly composed of LCNEC, alternative chemotherapy regimens should be explored. Regarding lung LCNEC, the adenocarcinoma-like subtype defined by the presence of *KRAS* or *STK11* mutations have higher response rates to the pemetrexed/platinum chemotherapy doublet [6,7,8,9,10]. Similarly, adenocarcinoma-like treatments are being compared to EP chemotherapy for digestive NEC, such as the FOLFIRINOX regimen, as part of the FOLFIRINEC trial (ClinicalTrials.gov Identifier: NCT04325425).

## 4. Materials and Methods

### 4.1. Patients

We retrospectively identified patients with pathologically confirmed digestive or lung NEN among all patients who received at least one cycle of EP chemotherapy in Beaujon Hospital (ENETS Centre of Excellence, Clichy, France) from 2006 to March 2020, whatever the tumor stage and with available histopathological material. We excluded patients whose morphological response could not be evaluated, for example, those without reevaluation imaging (early death) or those who received EP chemotherapy as adjuvant therapy following NEC surgical resection with curative intent. Patients with mixed neuroendocrine–non-neuroendocrine neoplasms (MiNEN) or grade 2 NEN (NET G2) were also excluded. 

All patients were treated with a standard protocol including etoposide (100 mg/m^2^/day on days 1–3) with cisplatine (45 mg/m^2^ on days 2–3 for digestive NEN or 25 mg/m^2^ on days 1–3 for lung NEN) or carboplatine (AUC 5 on day 1) every 3 weeks. Chemotherapy was always validated in NET expert multidisciplinary meetings. All patients underwent baseline contrast-enhanced CT scan within four weeks before EP. A biological and clinical evaluation was performed before each cycle of chemotherapy. The tumor response was assessed by contrast-enhanced CT performed every three to four cycles, or earlier in case of unexpected symptoms suggesting tumor progression.

### 4.2. Data Collected

We retrospectively collected data on epidemiology, clinical, biochemical and tumor features, pathology, treatment and toxicity. Anonymized data collection was performed for clinical records. The most common adverse events were recorded and graded according to the CTCAE 4.0 classification. All CT scans were centrally reviewed to measure the maximum variation in tumor size to determine the best morphological response to EP chemotherapy and the date of progression according to the Response Evaluation Criteria in Solid Tumors (RECIST), version 1.1. 

### 4.3. Histopathogical and Immunohistochemical Evaluation

Pre-chemotherapy tumor specimens were centrally reviewed for differentiation and immunohistochemistry (IHC) by two pathologists who specialized in NEN, together on a dual-head microscope. According to the 2019 WHO classification for digestive NEN and 2015 WHO classification for lung NEN, NEC were characterized by high-grade cytological criteria such as pleomorphisms, extensive necrosis, and prominent mitotic activity with high nucleo-cytoplasmic ratio. They were categorized as SCNEC (small or medium nuclei size, finely granular chromatin and inconspicuous nucleoli) or LCNEC (large nuclei, coarse chromatin and well-visible nucleoli). Conversely, NET were characterized by a low nucleocytoplasmic ratio, small to medium-sized nuclei, minimal pleomorphisms and minimal findings of necrosis. To simplify and homogenize the classification of NENs, we decided to classify all G3 NEN according to the 2019 WHO classification for digestive NEN [1], thus as G3 NET, LCNEC or SCNEC.

IHC was performed centrally with an automated staining system (Benchmark GX, Ventana, Tuscon, AZ, USA). Each slide was immune-labeled with monoclonal antibodies against Mib1 (1:100; Dako), Rb (1:500; clone 4H1, Cell signaling 9309), p53 (1:400; clone DO7, Agilent M700101) and p16 (Pure; clone E6H4; Ventana). Ki-67 was assessed while blinded to differentiation, as the percentage of positive tumor cell nuclei in at least 500 cells in the areas of highest staining density [40]. 

Rb, p53 and p16 staining were evaluated using a semiquantitative score, ranging from 0 to 300. Briefly, the percentage of positive tumor cells was multiplied by staining intensity graded from 0 to 3 (0: negative; 1: weak; 2: moderate; 3: strong). Because these neoplasms are highly proliferative, Rb expression was classified as inappropriate (Rb^inap^) if the score was 0 or appropriate (Rb^app^) in all other cases. p53 expression was classified as inappropriate (p53^inap^) for a score of 0 (no staining) or 300 (intense and homogenous staining probably corresponding to mutation in *TP53)* [14] or as p53^app^ in all other cases (heterogeneous staining). p16 expression was classified as low (p16^low^) in case of low or intermediate score (<200), or high (p16^high^) in cases with a score (>200). 

### 4.4. Statistical Analyses 

Quantitative data were described as medians (interquartile range 25–75) and compared using the Mann–Whitney test. Qualitative data were described as frequencies (percentages) and compared using the Chi-2 or Fisher tests, whichever was most appropriate. Correlations were explored using the Spearman test. 

The primary endpoint was the objective response rate (ORR), which was analyzed according to the pathological differentiation and IHC biomarkers. The most appropriate Rb cut-off value to identify responders was determined using the area under the receiver-operator curve (AUC) and the Youden index. Factors associated with the probability of an objective response were explored using logistic regression models. All clinically relevant, non colinear variables with a *p*-value <0.20 on univariate analysis were evaluated by stepwise backward regression models to identify the most relevant multivariate model, considering a maximum of 10 events per variable [41].

Secondary endpoints were progression-free survival (PFS) and overall survival (OS) in the NEC subgroup. For these analyses, patients with NET were excluded because their progression and prognosis are different from those with NEC. PFS was defined as the delay from the initiation of EP chemotherapy to disease progression or death from any cause, and patients were censored if there were no events at the final follow-up, or in case of a change in treatment with no confirmed progression. OS was defined as the time from EP therapy initiation until death from any cause, or patients were censored if they were alive at the final follow-up. PFS and OS were estimated using the Kaplan–Meier method and compared with the log-rank test. Factors associated with the risk of progression and with the risk of death were explored using the Cox proportional hazard univariate and multivariate models. A *p*-value <0.05 was considered to be statistically significant. All the analyses were performed using Prism© (version 6, Graphpad^TM^) and SPSS© (version 20, IBM^TM^) software.

### 4.5. Ethical Considerations

This study was performed according to the Helsinki convention. Data collection was anonymous following patient consent and institutional review board approval (IRB Paris-Nord University 00006477).

## 5. Conclusions

In conclusion, patients with G3 NET, LCNEC and SCNEC have different prognosis and responses to EP chemotherapy. We confirm the strong response to EP treatment in SCNEC, while its efficacy was moderate in LCNEC. Due to its poor efficacy in G3 NET, EP treatment should not be used for this indication. Among NEC, Rb < 150 was independently associated with a significantly increased probability of prolonged PFS (like p16) and an objective response. p53 did not strongly influence the response to EP therapy. However, treatments targeting NEC other than EP chemotherapy are limited. While our results must be confirmed in prospective studies, EP chemotherapy should be compared to organ-specific carcinoma-like treatments in NEC, stratified for Rb status in the future.

## Figures and Tables

**Table 1:** Characteristics of 89 patients with grade 3 neuroendocrine neoplasms (G3 NEN) treated with etoposide-platinum (EP) chemotherapy.

Baseline Characteristics	All (*n* = 89)
Age (years), median (IQR)	61.6 (54.2–68.6)
Male gender, *n* (%)	60 (67.4)
Hereditary syndrome, *n* (%)	1 (1.1)
Current or previous tobacco use, *n* (%)	49/71 (69)
Performance status, *n* (%)	
0–1	61/78 (78.2)
≥2	17/78 (21.7)
Primary NEN, *n* (%)	
Lung	37 (41.6)
Pancreas	27 (30.3)
Other digestive	25 (28.1)
Functioning syndrome, *n* (%)	1 (1.1)
Tumor stage, *n* (%)	
Localized or locally advanced	18 (20.2)
Liver metastases	61 (68.5)
Extra-hepatic metastases	43 (48.3)
2019 WHO classification, n (%)	
G3 NET	10 (11.2)
LCNEC	31 (34.8)
SCNEC	48 (54.0)
Ki67 (%), median (IQR) *	80.0 (57.5–90.0)
Biology, median (IQR)	
Albumin (g/L) **	32.0 (28.0–36.7)
Platelets (G/L) ***	288.0 (210.2–381.8)
Hemoglobin (g/dl) ***	11.9 (10.7–13.3)
Leukocytes (G/L) ***	8.3 (6.3–10.8)
Alkaline phosphatase (UNL) ***	1.3 (0.6–2.5)

* 1 missing value, ** 16 missing values, *** 3 missing values. IQR, interquartile (25–75) range; LCNEC, large-cell neuroendocrine carcinoma; NEN, neuroendocrine neoplasm; NET, neuroendocrine tumor; SCNEC, small-cell neuroendocrine carcinoma.

**Table 2:** Multivariate analysis of factors associated with objective response.

	OR (95%CI)	*p* Value
**Model 1: Rb^inap^**		
Lung primary (vs. other locations)	0.70 (0.21–2.38)	0.570
SCNEC (vs. NET or LCNEC)	8.89 (2.26–30.4)	0.001
Rb^inap^ (vs. Rb^app^)	1.70 (0.54–5.37)	0.368
Ki-67 (each additional 1%)	1 50.97–1.02)	0.550
**Model 2: Rb < 150**		
Lung primary (vs. other locations)	0.70 (0.20–2.41)	0.566
SCNEC (vs. NET or LCNEC)	7.83 (2.25–27.28)	0.001
Rb < 150 (vs. Rb score ≥ 150)	4.16 (1.11–15.53)	0.034
Ki-67 (each additional 1%)	0.99 (0.96–1.01)	0.309
**Model 3: p16^high^**		
Lung primary (vs. other locations)	0.71 (0.21–2.47)	0.602
SCNEC (vs. NET or LCNEC)	7.63 (2.19–26.57)	0.001
p16^high^ (vs. p16^low^)	1.61 (0.47–5.56)	0.449
Ki-67 (each additional 1%)	1 (0.97–1.02)	0.804

LCNEC, large-cell neuroendocrine carcinoma; NEN, neuroendocrine neoplasm; NET, neuroendocrine tumor; OR, odds ratio; SCNEC, small-cell neuroendocrine carcinoma.

**Table 3:** Multivariate analysis of factors associated with the risk of progression among patients with NEC.

	HR (95%CI)	*p* Value
**Model 1: Rb^inap^**		
Lung primary (vs. other locations)	1.39 (0.75–2.58)	0.301
SCNEC (vs. LCNEC)	0.54 (0.29–1.03)	0.06
Ki-67 (each additional 1%)	1.02 (1–1.03)	0.028
Extra-hepatic metastases (vs. absence)	1.04 (0.62–1.75)	0.87
Rb^inap^ (vs. Rb^app^)	0.57 (0.32–1.02)	0.058
**Model 2: Rb < 150**		
Lung primary (vs. other locations)	1.31 (0.71–2.42)	0.393
SCNEC (vs. LCNEC)	0.59 (0.31–1.1)	0.097
Ki-67 (each additional 1%)	1.02 (1.00–1.04)	0.014
Extra-hepatic metastases (vs. absence)	1.1 (0.66–1.84)	0.712
Rb < 150 (vs. Rb score ≥ 150)	0.37 (0.17–0.8)	0.012
**Model 3: p16^high^**		
Lung primary (vs. other locations)	1.59 (0.82–3.09)	0.167
SCNEC (vs. LCNEC)	0.54 (0.27–1.09)	0.086
Ki-67 (each additional 1%)	1.02 (1–1.04)	0.018
Extra-hepatic metastases (vs. absence)	1.19 (0.71–1.98)	0.518
p16^high^ (vs. p16^low^)	0.39 (0.19–0.79)	0.009

HR, Hazard ratio; 95%CI, 95% confidence interval; SCNEC, small-cell neuroendocrine carcinoma; LCNEC, large-cell neuroendocrine carcinoma; NEN, neuroendocrine neoplasm.

**Table 4:** Multivariate analysis of factors associated with the risk of death among patients with NEC.

	HR (95%CI)	*p* Value
**Model 1: Rb^inap^**		
Lung primary (vs. other locations)	1.61 (0.87–2.99)	0.131
SCNEC (vs. LCNEC)	0.69 (0.37–1.27)	0.230
Ki-67 (each additional 1%)	1 (1.00–1.02)	0.166
Extra-hepatic metastases	1.95 (1.12–3.25)	0.010
Rb^inap^ (vs. Rb^app^)	0.54 (0.31–0.95)	0.033
**Model 2: Rb < 150**		
Lung primary (vs. other locations)	1.56 (0.84–2.91)	0.157
SCNEC (vs. LCNEC)	0.74 (0.40–1.39)	0.350
Ki-67 (each additional 1%)	1.01 (1–1.02)	0.151
Extra-hepatic metastases	2.03 (1.21–3.41)	0.007
Rb < 150 (vs. Rb score ≥ 150)	0.50 (0.25–1.01)	0.053
**Model 3: p16^high^**		
Lung primary (vs. other locations)	1.55 (0.82–2.95)	0.179
SCNEC (vs. LCNEC)	0.79 (0.39–1.59)	0.507
Ki-67 (each additional 1%)	1.01 (1–1.02)	0.172
Extra-hepatic metastases	2.08 (1.24–3.49)	0.005
p16^high^ (vs. p16^low^)	0.52 (0.26–1.05)	0.067

HR, Hazard ratio; 95%CI, 95% confidence interval; SCNEC, small-cell neuroendocrine carcinoma; LCNEC, large-cell neuroendocrine carcinoma; NEN, neuroendocrine neoplasm.

## Data Availability

Some datasets generated during the current study are not publicly available but could be available from the corresponding author on reasonable request.

## Supplementary Materials

The following are available online at https://www.mdpi.com/2072-6694/13/4/643/s1, Figure S1: Study flowchart of the study, Figure S2: Distribution of the Rb score, according to NEN classification and RECIST-defined objective response to EP chemotherapy. Table S1: Pathological characteristics according to the primary NEN, Table S2: Treatment toxicity.
