# Supplementary material for: Biomarkers of Response to Etoposide-Platinum Chemotherapy in Patients with Grade 3 Neuroendocrine Neoplasms
Source: Cancers (Basel). 2021 Feb 5;13(4):643. doi: 10.3390/cancers13040643 (PMC7915900; doi:10.3390/cancers13040643)
Supplement: Supplementary file 1 [file cancers-13-00643-s001.pdf]

Figure S1. Study flowchart of the study.

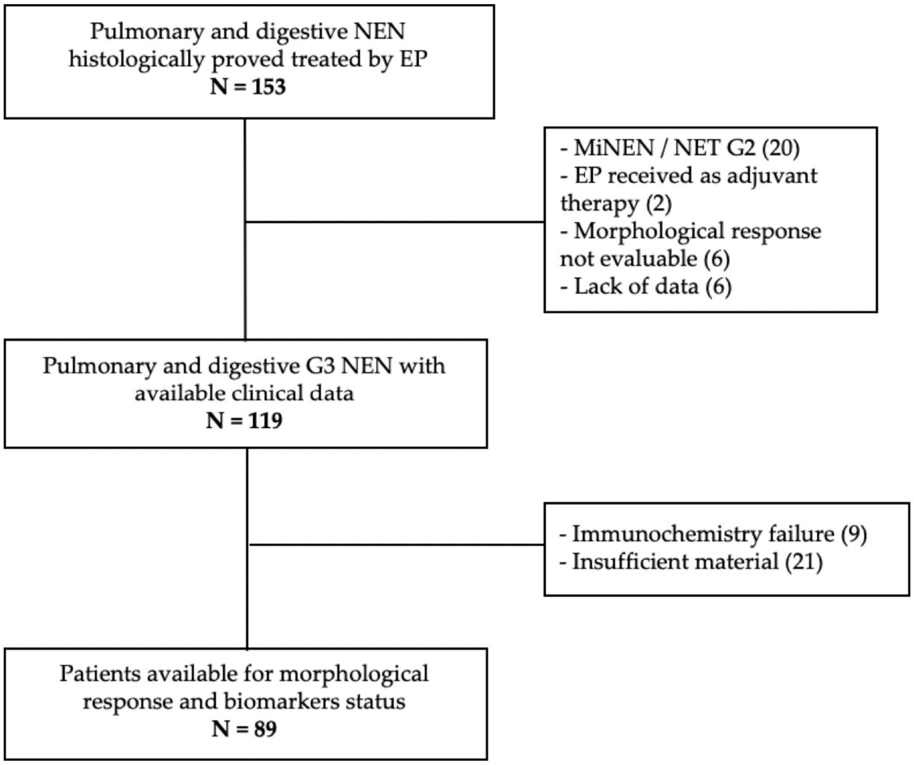

Figure S2. Distribution of the Rb score, according to NEN classification and RECIST-defined objective response to EP chemotherapy.

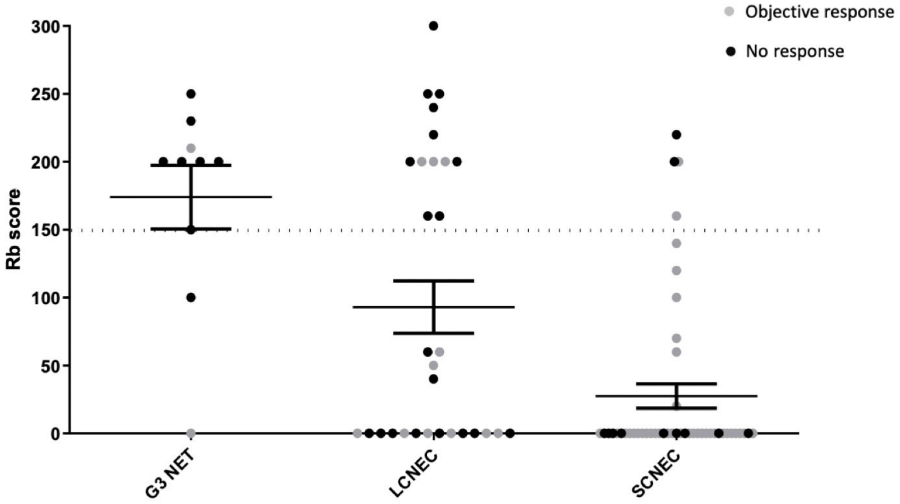

Table S1. Pathological characteristics according to the primary NEN.

	Lung NEN	Digestive NEN
Total, n (%)	37 (41.6)	52 (58.4)
2017-2019 WHO classification, n (%)		
G3 NET	0	10 (100)
LCNEC	7 (22.6)	24 (77.4)
SCNEC	30 (62.5)	18 (37.5)
Ki67 (%), median (IQR)	90.0 (80.0-92.5)	70.0 (39.0-90.0)
Biomarker expression, n (%)		
Rb^{inap}	29 (78.3)	22 (44.0)
Rb<150	33 (89.1)	30 (60.0)
$p16^{high}$	31 (86.1)	25 (49.0)
$p53^{inap}$	25 (67.6)	28 (57.1)

Table S2. Treatment toxicity.

n (%)	Grade 3-4
Fatigue*	7 (11.1)
Nausea and vomiting**	0 (0)
Neuropathy sensory**	4 (4.9)
Acute renal failure***	0 (0)
Leukopenia****	12 (13.9)
Neutropenia****	27 (31.4)
Thrombocytopenia****	7 (8.1)
Anemia****	8 (9.3)
